# Read the Advertisements

**Published:** 1896-01

**Authors:** 


### READ THE ADVERTISEMENTS.

Many of our readers have to dispense their own prescriptions and many of us are often puzzled to get the proper remedies, for certain conditions, nicely combined.

A glance over the advertising pages of THE JOURNAL will show you many elegant and excellent articles which meet your requirements, and which you can easily keep on hand, or, if in the city, prescribe with advantage to yourself and your patients. They are not patent medicines, but they are just as legitimate (more so) as the many foreign articles which are patented and which are constantly used. If you write to one of our advertisers, please mention THE JOURNAL. We wish you all a happy and prosperous year.

DR. CROTHERS states that of fifty-eight cases treated by him for inebriety, twenty-six were Keeley "graduates" and eleven others had been treated by some form of "gold cure."—*Ex.*

